# Supplementary material for: Fads2b Plays a Dominant Role in ∆6/∆5 Desaturation Activities Compared with Fads2a in Common Carp (Cyprinus carpio)
Source: Int J Mol Sci. 2023 Jun 26;24(13):10638. doi: 10.3390/ijms241310638 (PMC10341921; doi:10.3390/ijms241310638)
Supplement: Supplementary file 1 [file ijms-24-10638-s001.zip › ijms-2447930-supplementary.pdf]

Figure S1. Multiple sequences alignments of protein sequence from common carp Fads2a and Fads2b.

```
Fads2a    MGGGGQQTDR ITGTNARFST YTWEEVQKHT KSGDQWIVVE RKVYNVSQWV
Fads2b    MGGGGQQTDR IAGTNGRFGT YTWEEVQKHT KSGDQWIVVE RKVYNVSQWV
                                [cytochrome b5-like heme/steroid binding domain]

Fads2a    KRHPGGLRII GHYAGEDATE AFTAFHPDLP LVRKYMKPLL IGELEASEPS
Fads2b    KRHPGGLRII GHYAGEDATD AFHAFHPNIQ LVRKYMKPLL IGELEASEPS

Fads2a    QDRQKNAALV EDFRALRERL EAEGCFKTQP LFFLLHLGHI LLLEVIALML
Fads2b    QDRQKNGALV EDFRALRERL EAEGCFKTQP LFFILHLGHI LLLEAIALML

Fads2a    VWYFGTGWIN TAIVAVLMAT AQSQAGWLQH DFGHLSVCKS SRWNHLVHKF
Fads2b    LWYFGTGWIN TAIVSVILAT AQSQAGWLQH DFGHLSVFKN SRWDHLLHKF
                          [fatty acid desaturase domain]

Fads2a    VIGHLKGASA GWWNHRHFQH HAKPNVFKKD PDVNMLNMFV VGKVQPVEYG
Fads2b    VIGHLKGASA GWWNHRHFQH HAKPNIFKKD PDVNMLNAFV VGNVQPVEYG

Fads2a    VKKVKHLPYN HQHKYFFFVG PPLLIPVFFQ FQIFHNMVSH GLWVDLVWCI
Fads2b    VKKIKTLPYN HQHKYFFFIG PPLLIPVYFQ FQIIQNMITH GLWVDLMWCI

Fads2a    SYYVRYFLCY TQFYGLFWAV ILFNFVRFME SHWFVWVTQM SHIPMNIDYE
Fads2b    SYYVRYFLCY TQFYSVLWAV LLFNIVRFME SHWFVWVTQM SHIPMDIDYE

Fads2a    KHQDWLSMQL VATCNIEQSA FNDWFSGHLN FQIEHHLFPT MPRHNYWRAA
Fads2b    KHQDWLNMQL DATCNIEQSF FNDWFSGHLN FQIEHHLFPT MPRHNYWRAA

Fads2a    PHVRALCDKY GVKYQEKTLY GAFADIIRSL EKSGEIWLDA YLNK
Fads2b    PRVRALCDKY GVKYQEKGLY EAFVDIVRSL EKSGEIWLDA YLNK
```

The red letters represent different amino acids between two proteins. The predicted domains were indicated by blue (cytochrome b5-like heme/steroid binding domain) and green (fatty acid desaturase domain) boxes.

Figure S2. The 3D structure of common carp Fads2a, Fads2b and zebra fish Fads2.

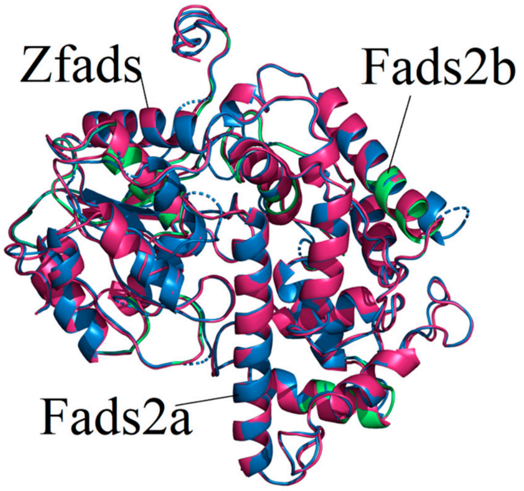

The 3D structure of common carp Fads2a (blue), Fads2b (green) and zebra fish Fads2 (Zfads, purple) protein of common carp predicted by homology modeling.

Figure S3. Characterization of desaturase activities of pYES2 without *fads2a* and *fads2b* cDNA inserts in transgenic yeast.

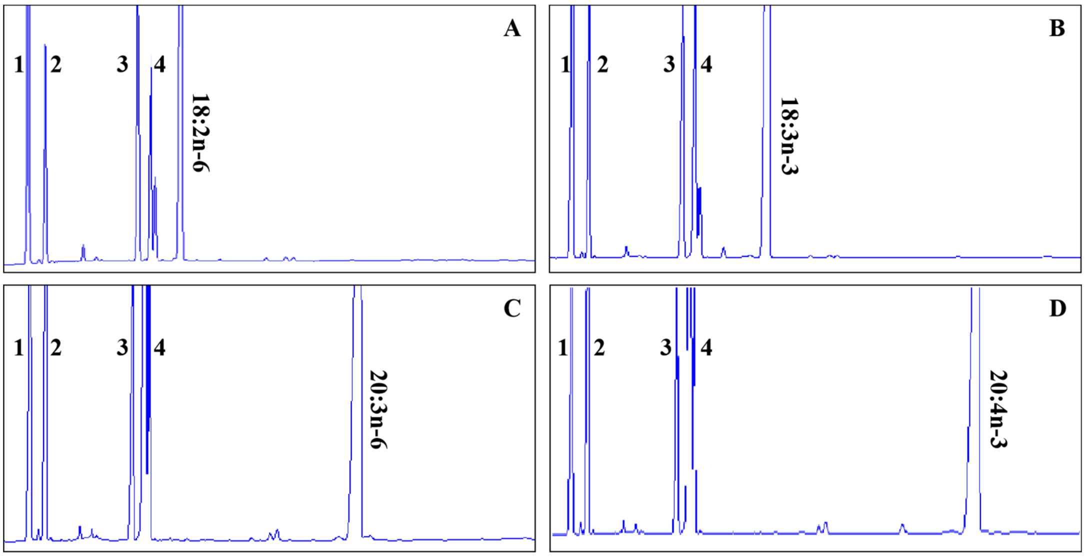

Characterization of desaturase activities of pYES2 without *fads2a* and *fads2b* cDNA inserts (negative control) in transgenic yeast (S. cerevisiae) towards grown in the presence of fatty acid substrates C18:2n-6 (A), C18:3n-3 (B), C20:3n-6 (C) and C20:4n-3 (D). Peaks 1 to 4 represent C16:0 (1), C16:1n-7 (2), C18:0 (3) and C18:1n-9 (4), respectively, which are the four main endogenous fatty acids of S. cerevisiae. The vertical axis displayed the flame-ionization detector response peak. The horizontal axis showed the peak retention time (min).

Figure S4. The modeling of common carp Fads2a and Fads2b.

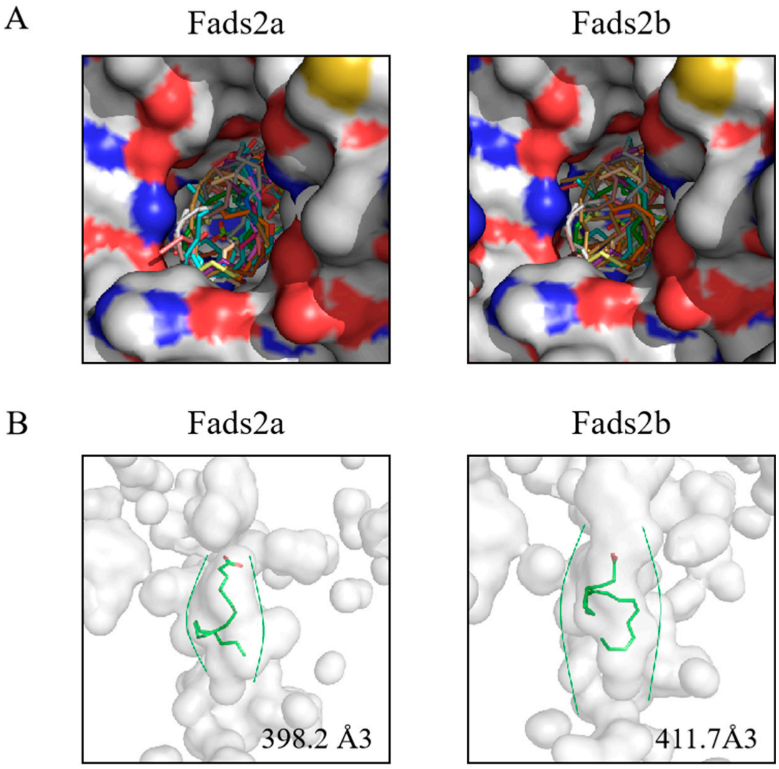

(A) Results of molecular docking analysis between 10 fatty acid small molecules and Fads2a/Fads2b proteins in Common carp. (B) The pocket niche space of Fads2a and Fads2b proteins.

Figure S5. Docking pose of the Δ6 and Δ5 substrates small molecules in Fads2a and Fads2b protein.

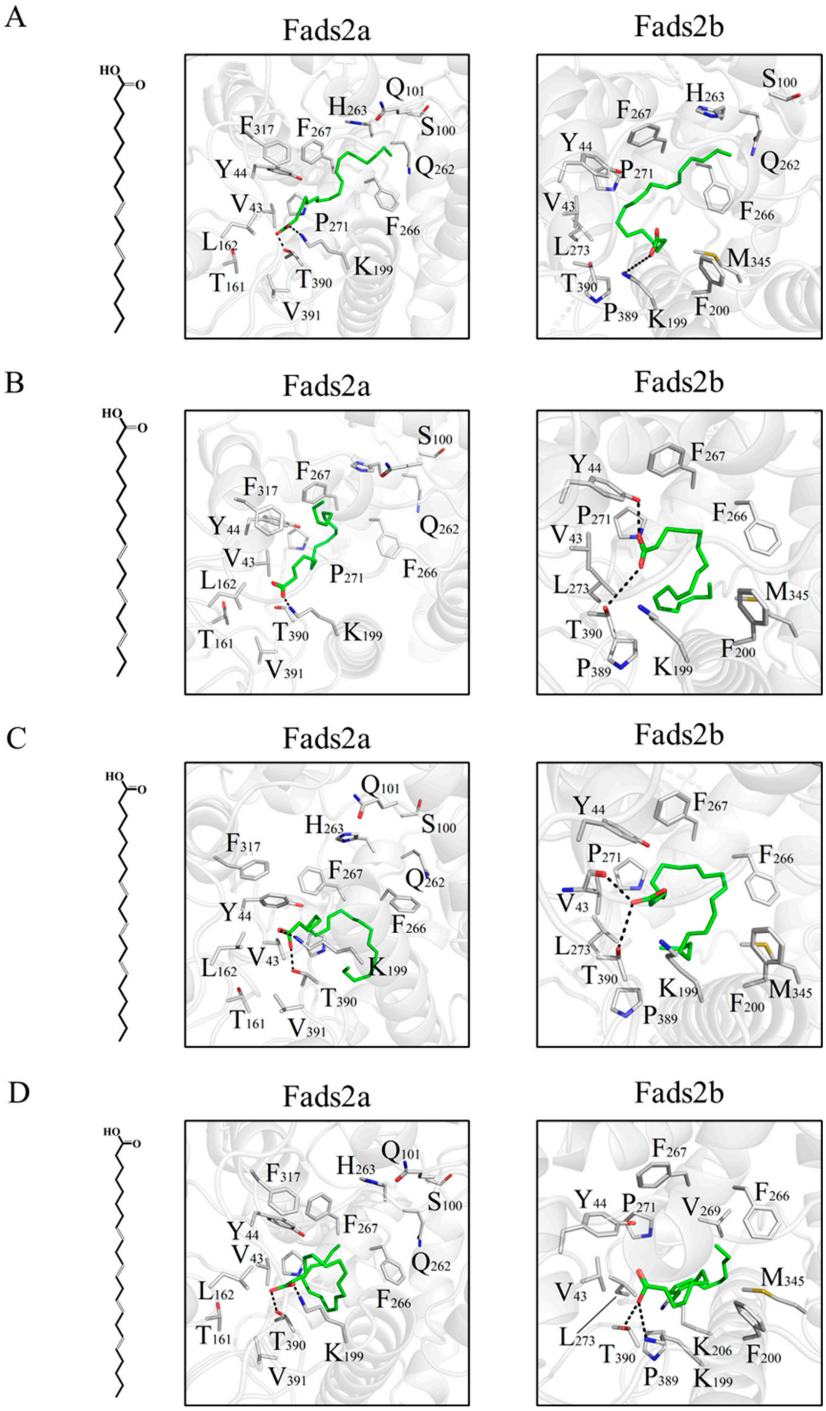

Docking pose of the Δ6 and Δ5 substrates small molecules (green color) C18:2n-6 (A), C18:3n-3 (B), C20:3n-6 (C) and C20:4n-3 (D) in the backbone peptide structure of Fads2a/Fads2b protein (gray color).

Table S1. Genbank accessions of proteins used in the phylogenetic analysis.

Species	NCBI Reference Sequence
Cyprinus carpio Fads2a	XP_042570977.1
Cyprinus carpio Fads2b	XP_042609343.1
Danio rerio	NP_571720.2
Homo sapiens	NP_004256.1
Mus musculus	NP_062673.1
Bos taurus	NP_001076913.1
Rattus norvegicus	NP_112634.1
Paralichthys olivaceus	XP_019933638.1
Lates calcarifer	XP_018537439.1
Salmo salar Fads2.1	NP_001165251.1
Salmo salar Fads2.2	NP_001165752.1
Carassius auratus Fads2.1	XP_026094349.1
Carassius auratus Fads2.2	XP_026057919.1
Sinocyclocheilus anshuiensis Fads2.1	XP_016298063.1
Sinocyclocheilus anshuiensis Fads2.2	XP_016324433.1
Larimichthys crocea	NP_001290292.1
Xiphophorus maculatus	XP_005807011.1
Poecilia formosa	XP_007556956.1
Oryzias latipes	XP_004069638.1
Oreochromis niloticus	XP_005470692.1
Trachinotus ovatus Fads2a	QBA19079.1
Trachinotus ovatus Fads2b	QBA19080.1
Lepisosteus oculatus	AYG96562.1
Siganus canaliculatus Fads2.1	ADJ29913.1
Siganus canaliculatus Fads2.2	ABR12315.2
Sparus aurata	AAL17639.1

Table S2. Fatty acid composition of common carp embryos at different developmental stages.

Fatty acids (μg/mg)	0 hpf	24 hpf	48 hpf	72 hpf	96 hpf	120 hpf
C14:0	4.381±0.045	4.483±0.024	4.293±0.013	3.988±0.029	3.387±0.025	3.485±0.010
C15:0	1.531±0.029	1.956±0.008	2.046±0.009	2.328±0.014	2.579±0.012	2.487±0.010
C16:0	288.359±1.594	307.884±0.970	290.446±0.619	300.984±2.157	306.498±2.038	311.828±1.222
C18:0	165.870±7.783	106.741±0.318	89.112±0.235	105.497±0.540	117.402±0.449	128.205±0.164
C20:0	0.373±0.022	0.866±0.007	0.864±0.006	1.300±0.001	1.492±0.003	1.444±0.001
Total saturated	460.514±8.446	421.931±1.258	386.760±0.867	414.096±2.732	431.358±2.514	447.449±1.393
C16:1n-7	13.052±0.407	16.960±0.034	18.462±0.052	15.269±0.101	12.704±0.164	11.085±0.133
C18:1n-9	127.868±3.311	188.633±0.242	208.082±0.360	200.989±4.527	185.883±4.515	168.859±4.599
C20:1	11.747±0.348	9.880±0.030	10.910±0.036	10.185±0.046	9.687±0.027	8.354±0.020
Total monounsaturated	152.666±3.713	215.473±0.258	237.455±0.390	226.443±4.388	208.274±4.330	188.298±4.462
C18:2n-6	117.338±0.580	98.128±0.302	107.496±0.232	88.560±0.535	68.218±0.359	62.426±0.494
C18:3n-6	9.396±0.259	23.592±0.245	14.486±0.254	14.118±0.471	16.776±0.438	20.267±0.345
C20:2n-6	12.431±0.294	11.224±0.047	11.952±0.025	11.391±0.038	10.877±0.018	10.100±0.013
C20:3n-6	31.989±0.973	18.250±0.073	19.638±0.031	18.066±0.142	16.635±0.056	15.310±0.043
C20:4n-6	94.506±2.512	95.851±0.282	100.394±0.340	101.399±0.788	109.438±0.878	108.951±0.576
C22:4n-6	10.941±0.431	9.847±0.122	10.906±0.040	10.782±0.046	11.164±0.122	10.975±0.090
C22:5n-6	22.259±0.909	19.980±0.180	21.869±0.096	22.323±0.149	24.670±0.294	25.216±0.213
Total n-6 PUFA	298.860±3.657	276.872±0.613	286.741±0.640	266.640±1.070	257.778±1.939	253.245±1.582
C18:3n-3	5.702±0.111	3.740±0.001	4.131±0.009	2.997±0.024	2.008±0.013	1.984±0.025
C18:4n-3	0.630±0.019	1.304±0.344	0.644±0.007	0.980±0.381	0.802±0.334	0.561±0.131
C20:3n-3	1.281±0.034	1.867±0.263	1.500±0.327	1.821±0.158	1.755±0.144	1.875±0.087
C20:4n-3	0.591±0.037	0.758±0.209	0.789±0.236	1.019±0.245	1.086±0.531	0.323±0.010
C20:5n-3	4.050±0.056	3.415±0.019	3.534±0.014	3.335±0.028	3.158±0.026	3.249±0.041
C22:5n-3	4.506±0.187	5.110±0.198	5.035±1.129	4.950±0.382	4.385±0.335	5.024±0.869
C22:6n-3	71.200±1.467	69.579±0.308	73.341±0.594	77.643±0.669	89.435±0.965	98.058±0.745
Total n-3 PUFA	87.959±1.740	85.772±0.858	88.974±1.607	92.746±1.090	102.629±0.284	111.075±1.507
Total PUFA	386.819±5.295	362.644±1.470	375.714±1.169	359.385±1.957	360.408±2.202	364.320±3.085

Table S3. Primers for ORF sequence amplification of common carp *fads2a* and *fads2b*

Primers	Sequence (5'~3')	Product length
fads2a-ORF-F	AGTCAGAGTTTGATCAGTTATGGG	1335 bp
fads2a-ORF-R	GAGAGGTTTTATTTGTTGAGGTACG	
fads2b-ORF-F	GAGTTTGATCAGCGATGGGC	1335 bp
fads2b-ORF-R	GAGGTTTTATTTGTTGAGGTACGC	

Table S4. Primers for RACE amplification of common carp *fads2a* and *fads2b*

Primers	Sequence (5'~3')	Product length
5'RACE-F	AAGCAGTGGTATCAACGCAGAGT	820 bp
5'RACE-*fads2a*-R	TGAGCATGTTGACATCCGGG	
5'RACE-F	AAGCAGTGGTATCAACGCAGAGT	951 bp
5'RACE-*fads2b*-R	TGATGGTTGTAAGGCAGCGT	
3'RACE-*fads2a*-F	TCTTTGGGTGGACCTCGTGTGG	1069 bp
3'RACE Outer Primer	TACCGTCGTTCCACTAGTGATTT	
3'RACE-*fads2b*-F	GTGCTTTGGGCGGTGCTTCTG	952 bp
3'RACE Outer Primer	TACCGTCGTTCCACTAGTGATTT	
3'RACE-*fads2a*-F	TCTTTGGGTGGACCTCGTGTGG	1069 bp
3'RACE Inner Primer	CGCGGATCCTCCACTAGTGATTTCACTATAGG	
3'RACE-*fads2b*-F	GTGCTTTGGGCGGTGCTTCTG	952 bp
3'RACE Inner Primer	CGCGGATCCTCCACTAGTGATTTCACTATAGG	

Table S5. Primers for Yeast expression vector pYES2.0 inserts

Primers	Sequence (5'~3')	Restriction site
pYES2-*fads2a*-F	CCCAAGCTTAGTCAGAGTTTGATCAGTTATGGG	Hind Ⅲ
pYES2-*fads2a*-R	CCGCTCGAGGAGAGGTTTTATTTGTTGAGGTACG	Xho I
pYES2-*fads2b*-F	CCCAAGCTTGAGTTTGATCAGCGATGGGC	Hind Ⅲ
pYES2-*fads2b*-R	CCGCTCGAGGAGGTTTTATTTGTTGAGGTACGC	Xho I

Restriction sites are underlined in primers.

Table S6. Primers for time and spatial RT-qPCR of common carp

Primers	Sequence (5'~3')	Product length
fads2a-qPCR-F	CCAGTGGGTGAAGAGACACC	113 bp
fads2a-qPCR-R	ATTTCCTCACCAGCGGAAGG	
fads2b-qPCR-F	GAAGAGACACCCCGGAGGAC	116 bp
fads2b-qPCR-R	GCGGCTTCATGTATTTCCTCAC	
β-actin-F	TGCAAAGCCGGATTCGCTGG	100 bp
β-actin-R	AGTTGGTGACAATACCGTGC	

Table S7. Primer for WISH probe amplification

Primers	Sequence (5'~3')	Product length
WISH-*fads2a*-F	GCCTTTGCGGACATCATTAGG	365 bp
WISH-*fads2a*-R	AATCACACGACTTTCAGGCA	
WISH-*fads2b*-F	AGGGCTTGTATGAGGCCTTTG	345 bp
WISH-*fads2b*-R	TTCCAGGTCATTCATGCACGTT	

Table S8. Primers for Dual-luciferase reporter vector pGL3-Basic inserts

Primers	Sequence (5’~3’)	Restriction site
fads2a-1-F（2.5K）	CTAGCTAGCGGGCAGGCTTCCAAGTCATA	Xho I
fads2a-2-F（2K）	CTAGCTAGCAGGTTTTTCACTGGAGGGCA	Xho I
fads2a-3-F（1.5K）	CTAGCTAGCATGTGTCCCTGCTTTGACGG	Xho I
fads2a-4-F（1K）	CTAGCTAGCTGTGCCGATATCACGTGTCC	Xho I
fads2a-5-F（0.5K）	CTAGCTAGCGCAACGTTTTGGGCATTACAG	Xho I
fads2a-promoter-R	CCGCTCGAGCAGATCATTCGAGTGCCACC	Hind III
fads2b-1-F（2.5K）	CTAGCTAGCGCACTGTGAGGACAGACAGAT	Xho I
fads2b-2-F（2K）	CTAGCTAGCAGTGAGCGAGTGCTGACATC	Xho I
fads2b-3-F（1.5K）	CTAGCTAGCCATGCAGGGCTGTGACCATT	Xho I
fads2b-4-F（1K）	CTAGCTAGCGTCATGAGCCAAGCTGATAAG	Xho I
fads2b-5-F（0.5K）	CTAGCTAGCGAAGGATGGTAAAATGCAACTG	Xho I
fads2b-promoter-R	CCGCTCGAGAGCGGAAGAGAGGCTCGC	Hind III

Restriction sites are underlined in primers.
